# Study on the Influence of Steel Fiber Distribution on the Mechanical Properties of Perfobond Leiste (PBL) Shear Connectors

**DOI:** 10.3390/ma16237387

**Published:** 2023-11-27

**Authors:** Yurui Zhang, Wenyuan Liao, Yuting Fang

**Affiliations:** College of Civil Engineering, Southwest Forestry University, Kunming 650224, China; 13678795973@163.com (Y.Z.); fangyuting@swfu.edu.cn (Y.F.)

**Keywords:** composite beam, PBL shear connector, steel fiber, finite element analysis

## Abstract

In order to study the influence of steel fibers on the mechanical properties of Perfobond Leiste (PBL) shear connectors and improve the utilization of steel fibers in this structure, four push-out test specimens and eight finite element numerical models were produced to study PBL-type shear connector specimens with different steel fiber blending amounts and blending forms. The results show that in this structure, when the blending amount of steel fiber was 0.5% to 1.5%, the ultimate bearing capacity of the specimen improved linearly, and the steel fiber helped to give full play to the performance of the PBL shear connector. The steel fibers distributed in the Z-direction have a significant impact on the mechanical properties of the PBL shear connector, and the steel fibers distributed in this direction have a significant effect on increasing the ultimate bearing capacity of the specimen. Steel fibers distributed in the Y-direction can greatly improve the plasticity of concrete. In addition, the effective action area of steel fibers is the triangular area from the bottom of the PBL shear connector to the two tops of the concrete.

## 1. Introduction

The PBL shear connector (Perfobond Leiste, PBL for short) is a shear connector composed of perforated steel plates through steel bars that connect concrete in the compression area and steel beams in the tension area [1,2,3,4]. Compared with traditional bolt shear connectors, PBL shear connectors have stronger shear resistance and greater stiffness and are widely used in steel–concrete composite structures in actual projects [5,6,7,8]. Leonhardt, Andra et al. [9] conducted a comparative analysis of PBL connectors and bolted connectors through experiments. The test results showed that PBL connectors have better stiffness and ductility and better fatigue resistance than bolted connectors. Cândido Martins et al. [10] used push-out tests to study PBL connectors and found that through-reinforcing steel bars in steel plate holes can significantly improve the shear load-bearing performance and ductility of the connectors. However, when PBL shear connectors are used to connect steel–concrete composite beams, premature failure of the concrete often occurs, resulting in the inability to fully exert the shear performance of the PBL shear connectors. If steel fibers with strong tensile strength are added to concrete to form Steel Fiber-Reinforced Concrete (SFRC), the mechanical properties of concrete will be greatly improved [11,12,13,14]. The improvement of concrete performance can bring out the shear resistance of PBL shear connectors, which can significantly improve the bearing capacity of steel–concrete composite beams [15].

The incorporation of steel fibers into concrete can effectively improve the various mechanical properties of concrete [16]. Naderi et al. [17] found that by adding straight-end, hook-end, and spiral steel fibers to concrete, the compressive toughness of concrete increased by 13.6%, 11.5%, and 2.6%, respectively. Zhang et al. [18] found that the fracture performance of concrete can be improved with an increase in the amount of steel fibers incorporated. When steel fibers are incorporated into concrete, the ultimate shear strength of composite beam connectors is significantly improved [19,20]. Steel fiber concrete is of great help in improving the mechanical properties of composite beams in this structure, but the utilization rate of steel fibers needs to be studied. The steel fibers inside concrete are affected by various conditions such as construction technology and component shape. Steel fibers are often unevenly dispersed in the matrix [21]. Uncertain dispersion will reduce the utilization rate. In addition, Zhu et al. [22] pointed out that the effective coefficient of randomly distributed steel fibers in a certain direction is 0.405, which means that the effective utilization rate of steel fibers is only approximately 40%. Existing research results have pointed out that the reasonable incorporation of steel fibers can more effectively improve the mechanical properties of steel fiber concrete. Ramiz et al. [23] found that improving the distribution of steel fibers can improve the bending performance of beams. Wu Kai et al. [24] found that the bonding performance of section steel and steel fiber concrete can effectively ensure that the two materials work together. Lu Dong et al. [25] found that under the same dosage conditions, appropriately increasing the elastic modulus of fibers can reduce the tensile strain of fibers and enhance the crack resistance of concrete. Yu Jiahuan et al. [26] found through experiments that adding steel fibers to concrete can transform the failure mode of concrete from brittle failure to plastic failure with a certain residual bearing capacity. Meng et al. [27] studied the effect of the steel fiber volume fraction on the flexural properties of concrete and concluded that as the fiber volume fraction increases, the crack propagation path becomes uniform. Zandi et al. [28] pointed out that the amount of fiber added to the concrete mixture is usually between 0.1% and 3.0%. When the steel fiber content is 2.0%, the stress of SFRC increases by 60.64% compared with the concrete without steel fiber. In summary, this article believes that changing the incorporation form of steel fibers is more efficient than increasing the overall content of steel fibers. Optimizing the incorporation form of steel fibers in this structure has a significant impact on future practical projects.

The above-mentioned research results mainly focus on the shear performance of shear connectors after steel fibers are completely incorporated into concrete, but there are few research results on optimizing the distribution form of steel fibers. Therefore, the research object of this article is steel fiber concrete composite beams with PBL shear connectors. Through push-out tests and finite element analysis, the influence of steel fiber distribution form on the mechanical properties of PBL shear connectors was studied. After comparative analysis, a reasonable amount of steel fiber inclusion and steel fiber distribution orientation were obtained, which provided a reference for practical engineering.

## 2. Materials and Methods

### 2.1. Material Properties

The concrete used in this study is of C40 grade. The H-shaped steel, PBL shear connectors, through-bars, and other steel materials used in the study are all Q235 grade. The steel fiber length is 15 mm, the diameter is 0.2 mm, and the tensile strength is 380.2 MPa. The mechanical properties of the materials used were tested in accordance with the “Standard for Test Methods of Physical and Mechanical Properties of Concrete [29]”. When the steel fiber content is 0.5%, 1%, and 1.5%, the compressive strength of more than three concrete cubes is tested and the average value is obtained. The obtained cubic compressive strengths of steel fiber concrete are 45.8 MPa, 47.4 MPa, and 48.3 MPa, respectively. The test obtained the compressive strength $f_{\mathrm{cu}}$ and elastic modulus $E_{C}$ of concrete and the yield strength $f_{y}$, ultimate strength $f_{u}$, and elastic modulus $E_{S}$ of steel. The results are shown in Table 1.

### 2.2. Specimen Design

**Figure 1:**
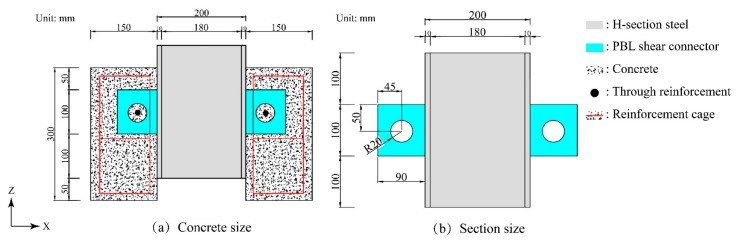
Specimen size diagram.

The design of the specimen was in accordance with the European specification EC4 [30], and the preparation of the specimen was carried out in accordance with the construction technology given by Zhang Xiuzhi et al. [31]. The fiber content and size number of the test specimen are shown in Table 2. Sample dimensions are shown in Figure 1.

### 2.3. Preparation and Loading

**Figure 2:**
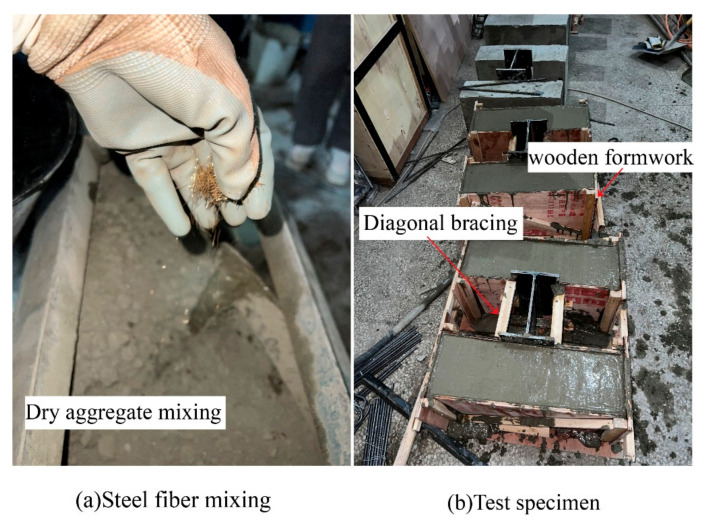
Specimen preparation.

When preparing steel fiber concrete, we consider the dispersion of steel fibers within the concrete. The dry aggregate is stirred before adding water, and the steel fibers are evenly added during the mixing process, as shown in Figure 2a. Considering the phenomenon of fiber hanging on the wall and sinking to the bottom, appropriate supplementation should be carried out according to the mixing ratio, and curing should be carried out indoors after preparation is completed (Figure 2b).

**Figure 3:**
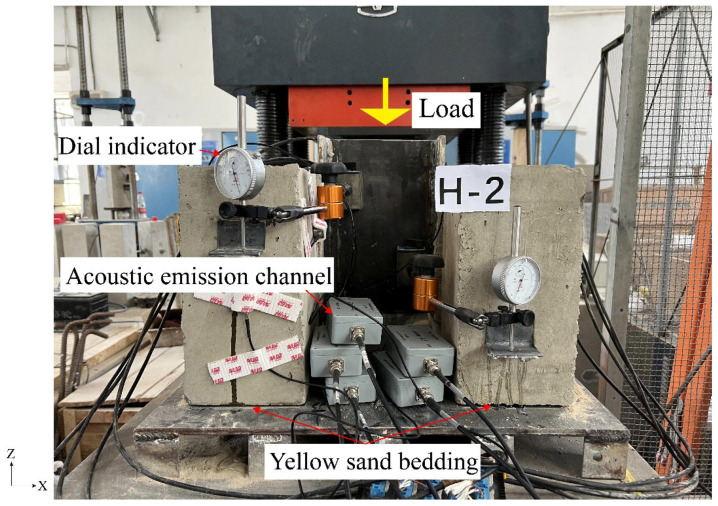
Specimen and loading device.

The test loading device and measuring point layout are shown in Figure 3. The test uses a YAW-1000KN microcomputer-controlled electro-hydraulic servo pressure testing machine for monotonic loading, and a yellow sand cushion is laid on the lower end of the concrete slab. In the early stage of test loading, force-controlled loading was used to reach 40% of the expected shear-bearing capacity, and then displacement-controlled loading was used. The loading rates of force control and displacement control were 0.5 kN/s and 0.03 mm/s, respectively. Displacement meters are arranged on the web and lower end of the steel beam of the specimen to measure the relative displacement between the loading end and the free end of the section steel and the test bench, respectively. After the acoustic emission data acquisition instrument performs sound source calibration, eight signal sensors are arranged horizontally and symmetrically on the left, right, and front of the concrete. After the waveform collection is completed, the data are played back and saved, and the channel coordinates and crack energy are extracted to observe the development of internal cracks in the concrete.

## 3. Finite Element Analysis

### 3.1. Build Finite Element Model

**Figure 4:**
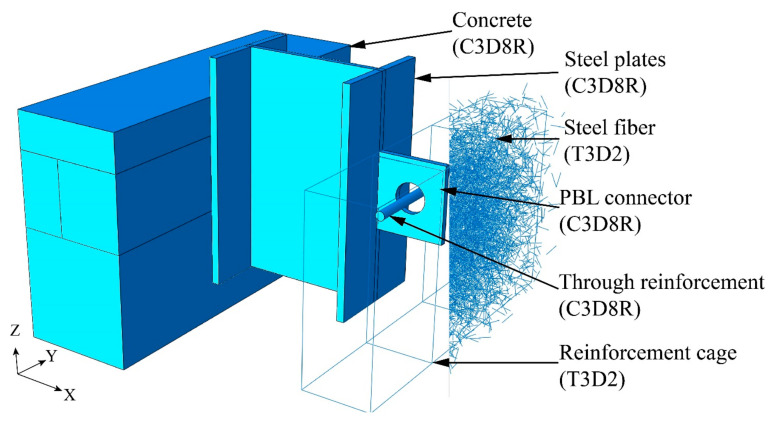
Finite element model.

ABAQUS was used for overall modeling. The layout size of the main stress-bearing parts, such as shear connectors and concrete tenons, was 2 mm, and the global layout size of the remaining parts was 12 mm. The stiffer and coarser mesh is usually set to the main surface, the tangential direction is frictionless, and the penalty direction is set to hard contact. The root of the shear connector and the steel flange is set in bound contact, and the concrete bottom surface is fully constrained. The contact friction coefficient is set to 0.6 according to the “General Code for Concrete Structures”. Concrete is considered to be damaged under tension and compression. When the maximum principal stress exceeds the compressive strength of concrete, it is considered to be a crushing failure. The steel fiber part is generated using PYTHON code, and the steel cage and steel fibers are embedded in the entire concrete model. The unit type is shown in Figure 4.

### 3.2. Material Properties and Constitutive Definitions

**Figure 5:**
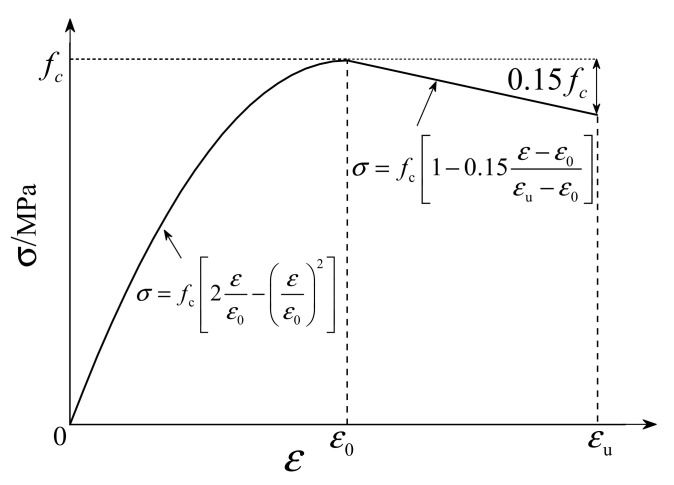
Stress–strain model of concrete.

The constitutive relationship of concrete under uniaxial compression adopts the formula suggested by Hognestad E [32], as seen in Equation (1). The rising section is a quadratic parabola, and the falling section is an oblique straight line, as shown in Figure 5. The compressive strength $f_{C}$ of concrete is 42.12 MPa, the density is 2420 kg/m³, the elastic modulus is 3.25 × 10^4^ MPa, the viscosity parameter is 0.0005, Poisson’s ratio is 0.2, the expansion angle is 30, the eccentricity is 0.1, and $fb_{0}/fc_{0}$ is 1.16. The constant stress ratio K between the tension meridian and the compression meridian is 0.667, the concrete peak strain $\varepsilon_{0}$ is 0.002, and the concrete ultimate strain $\varepsilon_{cu}$ is 0.0038.

(1) $$\sigma_{c}=\left\{\begin{array}{l} f_{c}\left[2\frac{\varepsilon }{\varepsilon_{0}}-{\left(\frac{\varepsilon }{\varepsilon_{0}}\right)}^{2}\right]\;\;\;\;\;\varepsilon \leq \varepsilon_{0}\\ f_{c}\left[1-0.15\left(\frac{\varepsilon -\varepsilon_{0}}{\varepsilon_{cu}-\varepsilon_{0}}\right)\right]\;\;\;\;\;\;\varepsilon_{0}<\varepsilon \leq \varepsilon_{cu} \end{array}\right.\;\;\;$$

**Figure 6:**
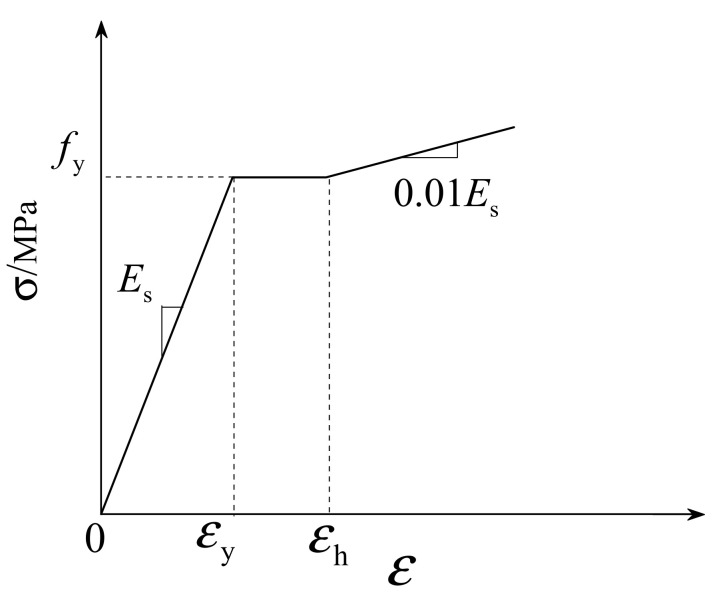
Stress–strain model of steel.

The multi-linear isotropic strengthening model for steel is shown in Figure 6, and its expression is shown in Equation (2). In this article, the tensile strength $f_{y}$ of the steel plate is 240.75 MPa, the density is 7890 kg/m³, Young’s modulus is 2.06 × 10^5^ MPa, $\varepsilon_{y}$ is the yield strain of the steel, $\varepsilon_{h}$ is the strengthening strain of the steel, and its value is $\varepsilon_{h}$ = 12$\varepsilon_{y}$, steel The strengthening modulus $E_{s}^{\prime }$ is 0.01 times the elastic modulus.

(2) $$\sigma =\left\{\begin{array}{l} E_{s}\varepsilon \;\;\;\;\;\;\;\;\;\;\;\;\;\;\;0<\varepsilon <\varepsilon_{y}\\ f_{y}\;\;\;\;\;\;\;\;\;\;\;\;\;\;\;\;\varepsilon_{y}\leq \varepsilon <\varepsilon_{h}\\ f_{y}+E_{s}^{\prime }(\varepsilon -\varepsilon_{h})\;\;\;\;\;\varepsilon \geq \varepsilon_{h} \end{array}\right.\;\;\;\;\;$$

## 4. Comparative Analysis of Tests and Finite Elements

### 4.1. Load-Slip Curve

**Figure 7:**
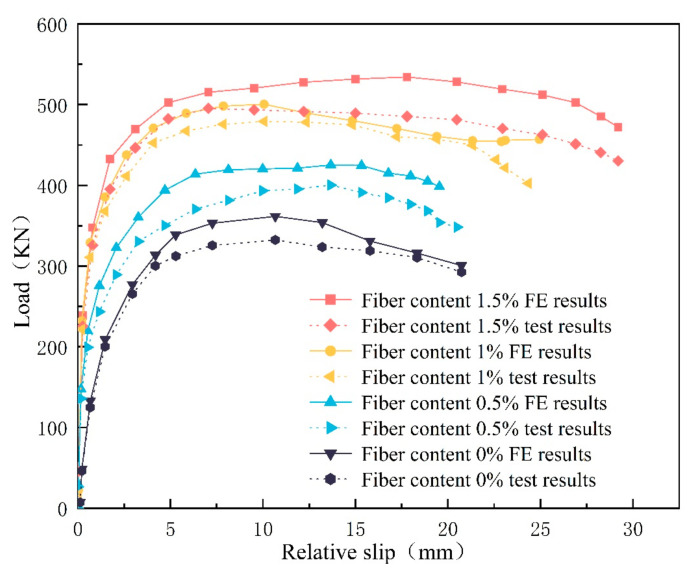
Finite element and test load–displacement curves.

The specimen load–slip curves and ultimate load capacity are shown in Figure 7 and Table 1. It can be seen that the incorporation of an appropriate amount of steel fibers can significantly enhance the ultimate load capacity of the specimens. Compared with A1, the ultimate load capacities of H1–H3 increase by 20.4%, 44.3%, and 49.1%, respectively, and the largest increase in the ultimate load capacity of the specimens is observed at the incorporation amount of 0.5–1.0%, which may be attributed to the fact that the parameters of steel fibers and coarse aggregates reached the optimum and exhibited flexural softening behavior. The increase in ultimate load capacity decreased significantly at 1–1.5% admixture, which may be due to the fact that the steel fibers tend to agglomerate around the concrete aggregate when the admixture is too large, resulting in uneven fiber dispersion, as well as flexural-hardening behavior, increased crack extension paths, and an increase in concrete defects.

The comparison shows that the errors between the experimental results of H1~H3 and the FEA results are 5.88%, 4.2%, and 7.3%, respectively, as shown in Table 3. By comparison, it is found that the error of A1 is the largest, which may be caused by the relatively large number of initial cracks and defects such as bubbles in ordinary concrete. H1, H2, and H3 have the reinforcement of steel fibers, and the elasticity and plasticity are enhanced. Fewer microscopic cracks are generated at the early stage of loading, and part of the energy is absorbed from crack expansion to fiber pull-out. The experimental process is closer to the finite element analysis process, and the errors of the ultimate load capacity of all the specimens are less than 10%, which indicates that the experimental results are in good agreement with the FEA results.

### 4.2. Specimen Damage

**Figure 8:**
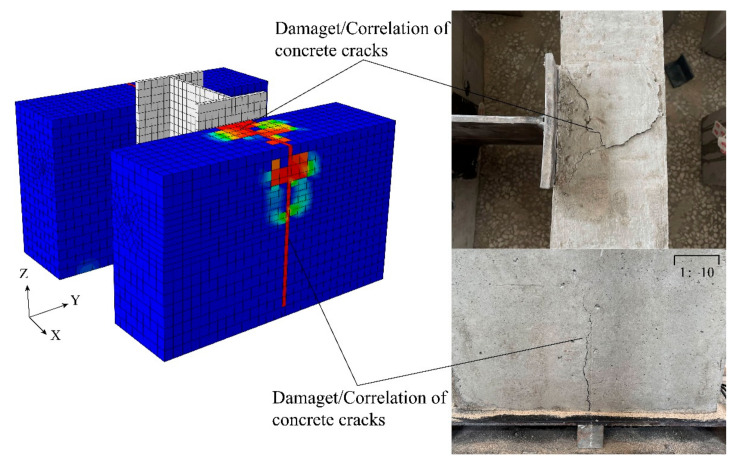
Concrete damage comparison.

The crack development and damage of H1 during the experiment are more consistent with the FEA results, as shown in Figure 8. When the displacement is loaded to 5 mm, due to the restraining effect of steel fibers on the concrete cracks, the tensile capacity of the concrete on the upper part of the PBL shear connectors is improved, and with the increase in shear, there is the phenomenon of stress concentration and there is a fragmentation of cracks in the area of the stress concentration, while the shear of the PBL shear connectors is sufficiently transferred to the concrete tenon. In the process of transferring the shear, the shear deformation of the PBL shear connectors and the compressive deformation of the concrete mortise together to absorb the energy until the destruction of the concrete mortise, the PBL shear connectors has produced a large deformation, and the outside of the concrete is also destroyed.

**Figure 9:**
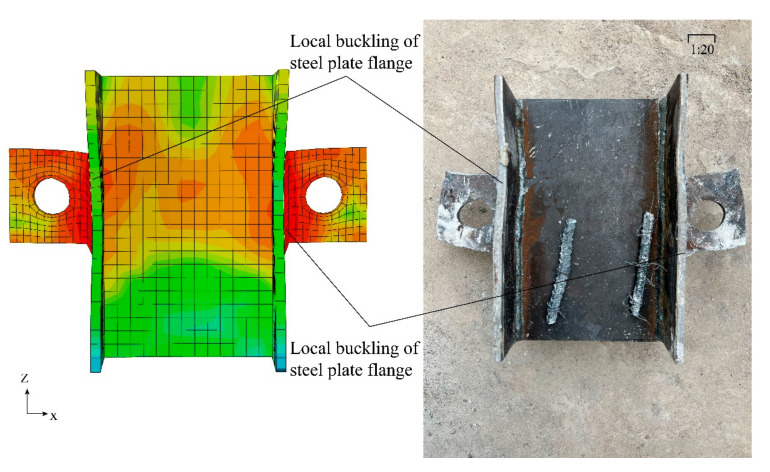
Comparison of stress and deformation of steel plates.

A comparison of the morphology of the H-steel taken out after the end of loading with the FEA results is shown in Figure 9. It can be seen that both the steel flange and the PBL plate produced buckling damage, which can be seen in the PBL shear connectors’ upper concrete damage, while the lower concrete compressive performance had not yet failed and a large number of shear forces flowed to the PBL shear connectors and concrete tenon. The existence of the concrete mortise results in the penetration of PBL shear connectors and the steel together, the lower part of the high-strength steel fiber concrete means that the PBL shear connectors give full play to the performance of the shear, the PBL shear connectors and the effective connection between the steel plate ensures that the residual shear moves back to the steel plate flange, and the steel flange and the PBL plate absorb some of the energy generated by the deformation of the buckling.

**Figure 10:**
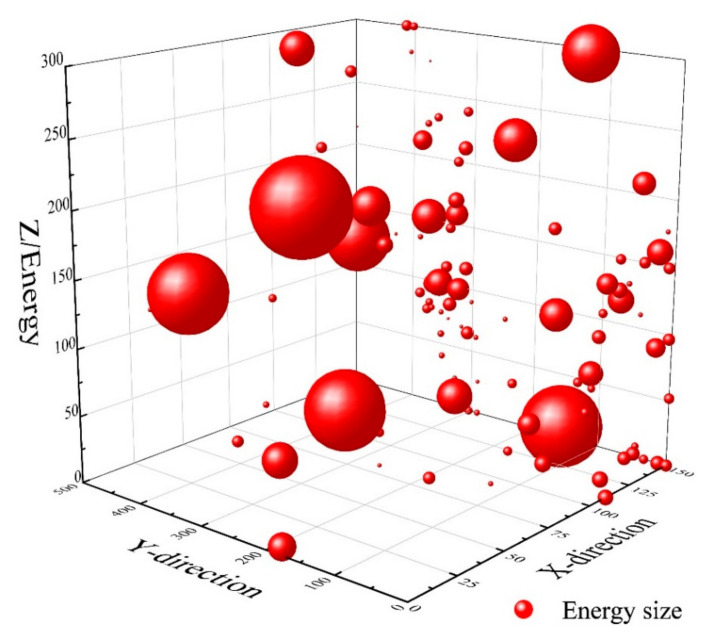
A1 specimen crack energy.

We then extracted full-waveform acoustic emission data collector data. The size of the sphere in the figure refers to the energy of the acoustic emission. This energy represents the detection of the occurrence and development of cracks within the concrete within a given period of time. In the figure, the X-, Y-, and Z-axes represent the internal space dimensions of the concrete, and the corresponding energy is the internal cracks in the concrete. The acoustic emission energy data of control group A1 without fiber blending is discrete, cracks develop randomly, and the energy difference is large, as shown in Figure 10.

**Figure 11:**
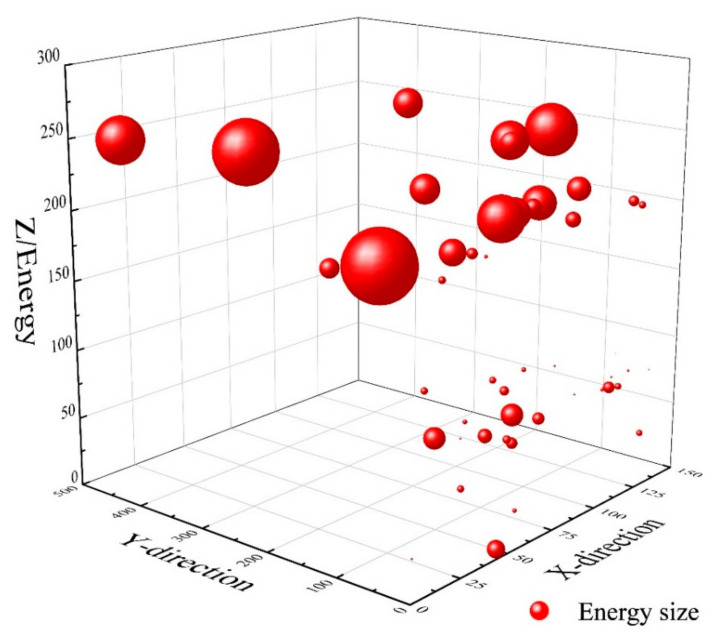
H2 specimen crack energy.

Most of the acoustic emission energy data detected by test group H2 with a fiber admixture of 1% is located in the upper part of the PBL joint. Considering that the upper part of the concrete is significantly stressed, the rest of the energy cracks appeared less, which shows that the steel fibers are effective in suppressing the concrete cracks. The crack energy diagram of H2 is shown in Figure 11.

## 5. Steel Fiber Distribution Impact Analysis

### 5.1. Parameter Design

Analyzing the above research, the mechanical properties of the combined beams are obtained when the steel fiber content is 0% to 1.5%. Considering the reliability of the parametric analysis, it is chosen to fix the steel fiber content as 0.5% and then design four specimens with different forms of steel fiber distribution according to the model dimensions and modeling methods designed in Section 2.1 and perform nonlinear finite element analysis to determine the most effective form of steel fiber distribution with the parameters as shown in Table 4. K1 is the control group with a steel fiber content of 0.5%, and the remaining three steel fiber distribution forms of different members K2~K4 are considered to be distributed from perpendicular to the shear flow direction and parallel to the shear flow direction, and the effects of steel fiber distribution forms on the shear performance of the specimens are analyzed.

### 5.2. Analysis of Results

The FEA results show that the ultimate load capacity of K2 decreases by 2.8% and the slip value increases by 20.3% compared to the control group K1. K3 decreases the ultimate load capacity by 18.7% and the slip value decreases by 33.2% compared to K1. K4 improves the ultimate load capacity by 6.7% and the slip value improves by 3.2% compared to K1. The data are shown in Table 5.

**Figure 12:**
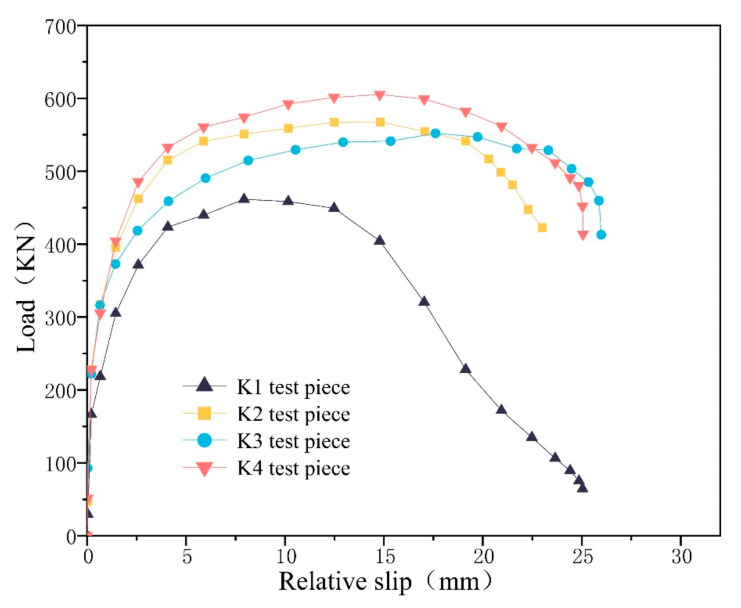
Finite element load–displacement curve.

The load–slip curves are shown in Figure 12. The K2 slip value of X-distributed fibers increased due to the fact that X-distributed fibers help to make up for the difference in the properties of the concrete at the two ends and can improve the ductility of part of the concrete. Therefore, under the same strength conditions, according to the actual situation, arranging the fibers according to the X-direction can achieve the same mechanical effect. The K3 slip and load capacity of Y-distributed fibers are both reduced because the Y-distribution of fibers is basically parallel to the direction of shear transmission, which fails to play a force advantage, and the concrete around the PBL shear connectors is more seriously damaged. The K4 slip and load capacity of Z-distributed fibers are both improved, and the utilization of steel fibers is the highest because the distribution direction of K4 steel fibers is approximately perpendicular to the direction of shear flow, which can exert good tensile capacity and is conducive to the transfer of shear.

**Figure 13:**
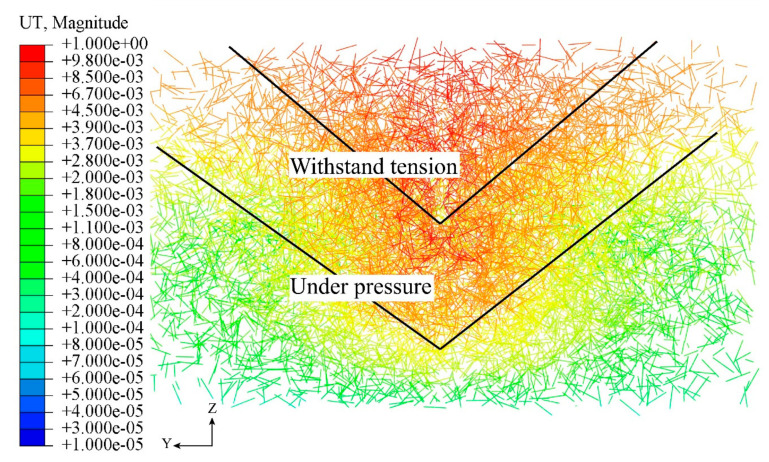
Steel fiber unbalanced torque.

In the reinforced concrete specimens undergoing pull-out tests, the form of load-bearing steel fibers inside the concrete goes through different stages. Taking H1 as an example, the steel fibers are in the elastic-plastic stage, where the fibers are constrained by friction and show energy-consuming characteristics of flexural and tensile resistance, and the deformation energy is generated slowly. As the load continues to increase, the concrete cracks begin to increase gradually, the internal energy begins to accumulate, the steel fibers are gradually pulled out, and the concrete begins to fracture, resulting in an energy dissipation effect, and finally, the concrete slab of the specimens is destroyed. The fiber unbalanced torque UT values of the specimen are shown in Figure 13. It can be observed that the unbalanced torque occurs mainly in the compressed concrete region at the lower part of the PBL shear connectors and then extends from the ends of the penetrating steel bars to the two tops of the concrete.

**Figure 14:**
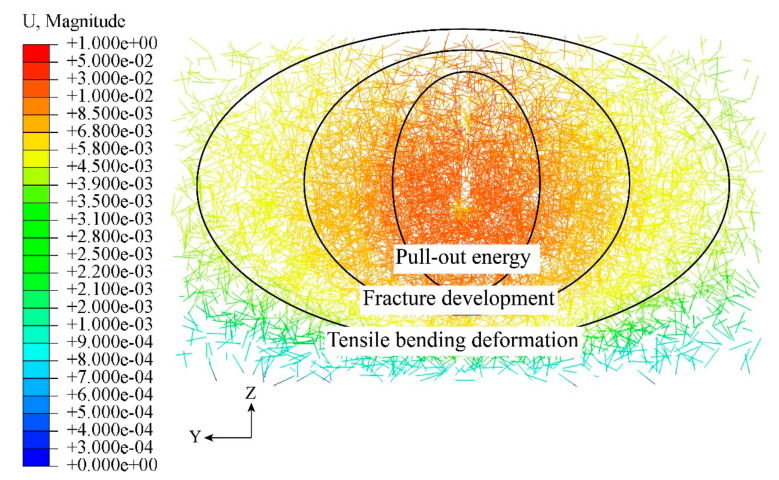
Steel fiber displacement.

The displacement U of the steel fiber inside the concrete is shown in Figure 14. From the figure, it can be seen that the penetrating reinforcement can share part of the shear with the upper concrete, which means the upper concrete is subjected to a larger load and significantly displaced. And the pull-out energy dissipation of the fibers is mainly distributed on both sides of the PBL shear connectors and in the upper and lower concrete, which indicates that K4, the type of fiber distribution, can be utilized to the maximum extent.

### 5.3. Shear Force and Flow Direction

**Figure 15:**
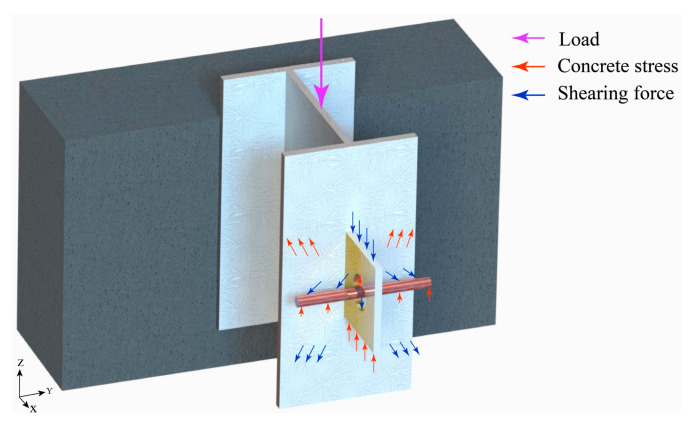
Shear flow direction.

Combining the experimental results and FEA results, as well as referring to the load–displacement curve shear value calculation method proposed by Zheng [33] in the pull-out test, it is concluded that the shear values of the four specimens are 517 kN, 472 kN, 428 kN, and 539 kN, respectively, with a fiber content of 0.5%, and the flow of the specimen shear is shown in Figure 15.

**Figure 16:**
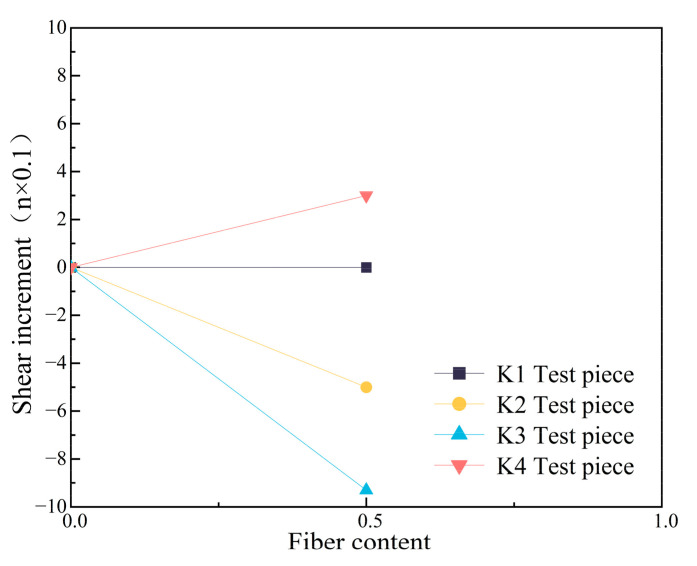
Steel fiber rate curve.

Based on the shear values derived above, comparing K1, the difference in shear values for K2 with an X-direction fiber distribution is 45 kN, for K3 with a Y-direction fiber distribution is 89 kN, and for K4 with a Z-direction fiber distribution is 22 kN, and the result is obtained by multiplying the difference in shear values by 0.1. Figure 16 shows the results when the steel fiber content is 0.5%, where the X-axis is the fiber content and the Y-axis is the shear increment. It provides a reference for the shear stiffness trend of the specimens after the increase in steel fiber content.

**Figure 17:**
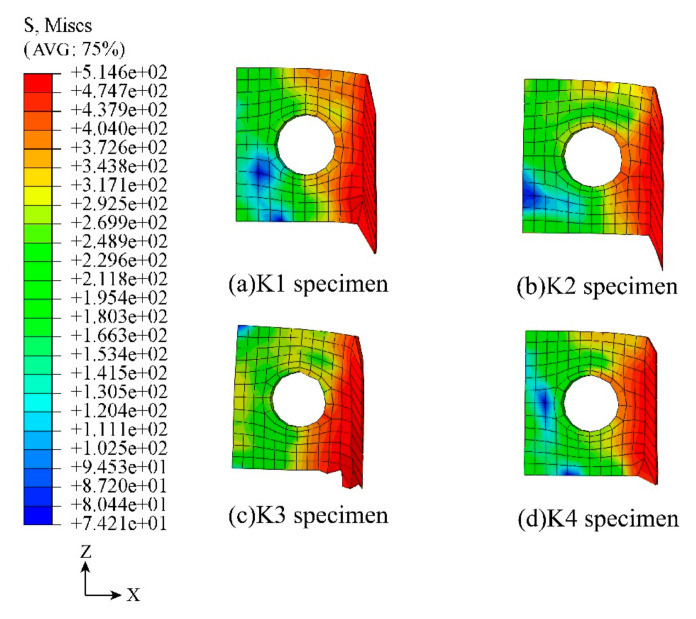
Deformation diagram of PBL connector.

The FEA results of the PBL shear connectors for the four specimens are observed and analyzed, and it is found that the stress concentration of PBL shear connectors in different content forms occurred on the side welded to the flange of the steel plate. The PBL shear connectors in K1 and K2 have similar deformation and stress concentrations, and the difference between the ultimate load capacity of these two specimens is small. The deformation of the specimens is shown in Figure 17.

The deformation of the PBL shear connectors of K3 and K4 is shown in Figure 17c,d. Observation of K3 shows that the Y-distributed fibers are parallel to the shear flow direction, and the effective fibers are distributed on both sides of the PBL shear connectors. Moreover, the lack of sufficient fiber reinforcement in the upper and lower parts of the concrete leads to the accumulation of shear forces conducted by the steel plate flanges here so that the PBL shear connectors are subjected to the imbalance reaction force to produce local buckling. Observation of K4 shows that the best performance of deformation control is obtained for the PBL open-cell plate, where the shear force is transferred in a more rational manner with this form of fiber distribution. Unlike the case of PBL where the shear connectors are directly in the concrete, the concrete is more uniformly stressed under the effective fibers.

**Figure 18:**
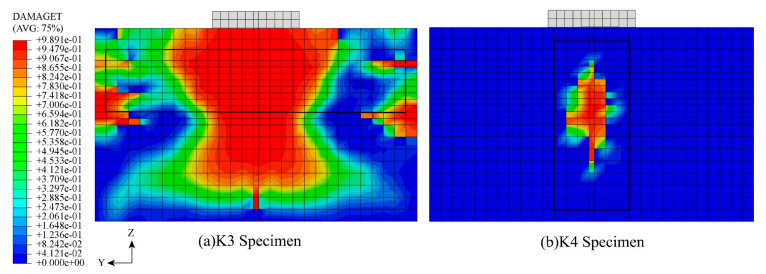
Concrete damage of K3 and K4 specimens.

The shear difference between K3 and K4 is the largest, and the damage cloud diagram of the specimens is shown in Figure 18. From the figure, it can be seen that the concrete damage area of the K3 specimen is larger. As the upper concrete is pulled to pieces, the concrete damage extends to the left and right ends, from which it can be seen that the fiber distribution parallel to the shear direction is insufficient to inhibit the concrete cracks. The steel fibers of specimen K4 are perpendicular to the shear direction, which has the best inhibition effect on the cracks and makes the transmission of shear in the specimen more uniform.

## 6. Conclusions

In this paper, the mechanical properties of PBL shear connectors in steel fiber concrete were investigated based on experimental and finite element modeling. By push-out tests, it could be found that the mechanical properties of the specimens were significantly enhanced when the steel fiber content was 0.5–1.5%. The experimental results were verified using ABAQUS finite element software. Synthesizing the above research methods, the following conclusions were drawn:

The addition of an appropriate amount of steel fibers could effectively improve the mechanical properties of concrete around the PBL shear connectors so that the shear performance of the PBL shear connectors could be brought into full play. There was a significant increase in the ultimate load capacity of the specimens with a steel fiber content of 0.5–1.0%, while the increase in the ultimate load capacity slowed down with a content of 1–1.5%.

In the case of the same fiber content, when the steel fibers were distributed in the X-direction, the ductility of the specimens was increased, while the load capacity was reduced. When distributed in the Y-direction, the load capacity and ductility of the specimens were reduced at the same time, and the utilization rate of the fiber was the lowest. When distributed in the Z-direction, the load capacity and ductility of the specimens were increased at the same time, and the fiber utilization rate was the highest. Therefore, the Z-distribution of steel fibers is the most advantageous for the PBL shear connectors.

During the shear transfer, the steel fibers performed most effectively in the triangular region formed by the lower part of the PBL shear connectors and the two tops of the concrete.

The energy dissipation mode of steel fibers around the PBL shear connectors was mainly pull-out energy dissipation, so in actual engineering, it can be mixed with end-hooked steel fibers to enhance the pull-out resistance of the specimens.

## Figures and Tables

**Table 1:** Material properties.

Materials	$f_{\mathrm{cu}}$/MPa	$E_{C}$/MPa	$f_{y}$/MPa	$f_{u}$/MPa	$E_{S}$/MPa
C40	42.12	3.25 × 10^4^	-	-	-
Q235	-	-	240.75	377	2.06 × 10^5^

**Table 2:** Specimen size data.

Number	Concrete Size/mm	H-Shaped Steel Dimensions/mm	PBL Size/mm	Fiber Content
A1	500 × 150 × 300	90 × 100	90 × 100; R = 20	0%
H1	500 × 150 × 300	90 × 100	90 × 100; R = 20	0.5%
H2	500 × 150 × 300	90 × 100	90 × 100; R = 20	1%
H3	500 × 150 × 300	90 × 100	90 × 100; R = 20	1.5%

**Table 3:** Comparison of test and finite element data.

Specimen No.	Experimental Ultimate Bearing Capacity/kN	Finite Element Ultimate Bearing Capacity/kN	Slippage/mm	Error/kN	Error Ratio
A1	332	361	16.7	29	8.03%
H1	400	425	20.5	25	5.88%
H2	479	500	24.9	21	4.2%
H3	495	534	29.2	39	7.3%

**Table 4:** Finite element test specimen size and number.

Specimen No.	Fiber Orientation	Distribution Size/mm	Fiber Content
K1	-	90 × 200 × 200	0.5%
K2	X	150 × 200 × 200	0.5%
K3	Y	90 × 500 × 200	0.5%
K4	Z	90 × 200 × 300	0.5%

**Table 5:** Bearing capacity and slip value.

Specimen No.	Ultimate Bearing Capacity/kN	Slippage/mm	Increment/kN	Amplification
K1	567	18.7	-	-
K2	551	22.5	−16	−2.8%
K3	461	12.5	−106	−18.7%
K4	605	19.3	38	6.7%

## Data Availability

Raw/processed data can be found in the article. Some of the data is not available as research is still ongoing.
